# Circular Utilization of Coffee Grounds as a Bio-Nutrient through Microbial Transformation for Leafy Vegetables

**DOI:** 10.3390/life14101299

**Published:** 2024-10-14

**Authors:** Hasan Ozer, Naime Ozdemir, Asude Ates, Rabia Koklu, Sinem Ozturk Erdem, Saim Ozdemir

**Affiliations:** 1 Department of Environmental Engineering, Faculty of Engineering, Sakarya University, Esentepe, 54187 Sakarya, Turkey; hasanozer@sakarya.edu.tr (H.O.); rkoklu@sakarya.edu.tr (R.K.); saimo@sakarya.edu.tr (S.O.); 2 Department of Horticulture, Faculty of Agriculture and Natural Science, Bilecik Seyh Edebali University, 11230 Bilecik, Turkey; naime.ozdemir@bilecik.edu.tr (N.O.); sinem.erdem@bilecik.edu.tr (S.O.E.)

**Keywords:** poultry litter biochar, starter inoculation, plant nutrients, spinach, garden cress

## Abstract

This study explores the production of bio-nutrients from bioactive compound-rich spent coffee grounds (SCG) and biochar (BC) through composting after inoculation with a biological agent and its impact on the growth performance of garden cress and spinach. The SCG was composted with six doses of BC (0, 5, 10, 15, 20, and 25%). The compost with 10% BC exhibited the best maturity, humification, and phytotoxicity index values of dissolved organic carbon (DOC), humification index (E4/E6), and germination index (GI). A metagenome analysis showed that compost starter enhanced the bacterial community’s relative abundance, richness, and diversity in SCG and BC treatments. This improvement included increased *Patescibacteria*, which can break down noxious phenolic compounds found in SCG and BC. The BC enriched the compost with phosphorus and potassium while preserving the nitrogen. In plant growth experiments, the total chlorophyll content in compost-treated garden cress and spinach was 2.47 and 4.88 mg g^−1^, respectively, which was significantly greater (*p* ≤ 0.05) than in unfertilized plants and similar to the plants treated with traditional fertilizer. Overall, the results show that the compost of SCG + BC was well-suited for promoting the growth of garden cress and spinach, providing adequate nutrients as a fertilizer for these leafy vegetables.

## 1. Introduction

In today’s world, there is a significant amount of disposable bio-waste that must be effectively managed to align with the United Nations Sustainable Development Goals. Failing to do so could lead to considerable environmental damage and loss of valuable nutrients [1]. Circular economy models offer an array of valorization methods to mitigate the adverse effects of bio-waste by keeping resources in the economy for as long as possible and minimizing waste. These approaches enable the reutilization of resources, thus creating additional value [2]. One widely used biotechnological method is composting, which involves converting various organic raw materials, including harmful compounds found in spent coffee grounds (SCG), into nutrient-rich biofertilizers through a diverse and beneficial microbial community. This biofertilizer can then be reused in crop production, promoting carbon neutrality [3] and supporting the transition to recyclable technology and domestic resource security [4].

Spent coffee grounds (SCGs) and the thermochemical process by-product biochar are the latest of decades of research into the full or partial replacement of peat with other organic and inorganic materials [5]. Both SCG and biochar are sterile to microbes due to high temperature production and contain stable carbon and plant nutrients, making them an interesting material for research topics from an agri-environmental perspective [6]. Similar to other bio-wastes, SCG is mainly disposed of in landfills and thus poses a threat to the environment as it contains useful plant nutrients and chemical compounds of biotechnological interest, such as organic acids, tannins, and phenolic compounds [7]. Although SCG contains significant amounts of plant nutrients, the direct use of SCG for soil improvement is associated with limitations due to its fine nature, low C/N ratio, and high moisture content, such as clogging of soil porosity, hydrophobicity of soil aggregates, phytotoxicity, and nutrient imbalance for crops [8] even for plants lower in acid. In this context, bioconversion through composting was proposed as a safe practice to improve challenges of SCG in order to fully exploit its nutritional value and soil mitigating potential.

Combining acidic spent coffee grounds (SCGs) with alkaline biochar through composting could present a sustainable solution for utilizing high-nutrient waste in the preparation of bio-nutrient or growing media constituents. Composting is a safe and straightforward process that contributes to the circular economy model and efficient waste management [9], enabling renewability in crop production. However, raw SCG and biochar do not possess the necessary characteristics for ideal growing media due to their structural, biological, and chemical limitations [10]. For example, SCG contains various bioactive compounds, such as polyphenols, tannic acid, organic acids, lipids, and caffeine, which can be phytotoxic to certain plants [11]. Similarly, although biochar offers benefits such as carbon sequestration, acting as a nutrient source and retaining nutrients in the soil–plant matrix, its alkaline nature restricts its direct use, potentially causing toxic effects or nutrient imbalances, thus requiring conditioning before use [12]. By leveraging the potentially favorable chemical composition of SCG and the porous physical properties of biochar, favorable composting conditions can be created, providing a natural organic nutrient source for potted crops [13]. Composting is a microbial-driven process that rapidly breaks down readily available organic matter into humus, yielding several organic acids that are essential for optimizing both materials for crop fertilization.

In recent years, biochar was found to be capable of enhancing biological processes, increasing bacterial diversity by providing a promising microbial environment for several forms of aerobic and anaerobic degradation, particularly for composting microorganism [14]. In this regard, the use of biochar as a microbial-enhancing tool would be a highly desired for rapid composting processes, where noxious bioactive compounds present in SCG and BC can be converted into nonphytotoxic plant nutrients that are safely applicable to cultivated crops.

Setting up new circular economic approaches in waste management in urban ecosystems by valorizing by-products of the agro-food industry as a secondary feedstuff for the crop production cycle is crucial for the sustainability of the urban farming and the environment. The present research aimed to examine the feasibility of producing a nutrient-rich bio-nutrient source by composting spent coffee grounds with poultry litter biochar and evaluate their potential use as a nutrient resource in pot growing media for leafy vegetable production in an urban vertical growing system setting. It was hypothesized that composting SCG with biochar would be a suitable growing media option for nutrient-rich horticultural substrates. To test this hypothesis, pot experiments were performed in a vertical growing system using two leafy vegetable crop garden cresses and spinach employing different percentages of compost to replace peat-based growing substrates.

## 2. Materials and Methods

### 2.1. Composting and Growing Media Preperation

**Figure 1:**
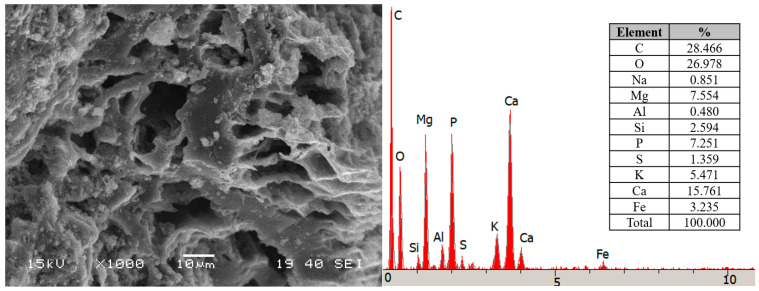
SEM image and EDS analysis of poultry litter biochar.

The raw materials used in the compost experiments consisted of spent coffee grounds collected from the coffee shop market Sakarya, Turkey. Biochar was provided by a commercial gasification plant (Bolu province, Turkey) that uses poultry litter collected from a nearby poultry farm with wood shavings/saw dust bedding material for chicks. The gasification plant process wastes at 600 °C with a residence time of 3.5 h to produce mainly syngas for electricity generation. At the same time, several byproducts are generated, such as biochar. The main characteristics of SCG and BC based on elemental analyzer (LECO CHN628, St. Joseph, MI, USA) in accordance with ASTM D5373-14 guidelines are presented in Table 1. The pH, total nitrogen content (TN), and total organic carbon (C) of SCG were 5.61, 2.16, and 52.06%, respectively. The main physical characteristics of poultry litter biochar were as follows: alkaline in nature, particles of 2–4 mm, the Brunauer–Emmett–Teller (BET) surface area of 13.58 m^2^ g^−1^ and total pore volume of 1.55 cm^3^ g^−1^, and average pore diameter 6.21 Å) (Figure 1). It is rich in non-volatile essential nutrients for plant fertilizer such as P and K (Table 1, Figure 1).

The experiment used small 6 L insulated reactors (with a diameter of 27.5 cm and a height of 17 cm) for 45 days. Different compost treatments were mixed with BC at varying percentages (5%, 10%, 15%, 20%, and 25% dry basis *v*/*v*) to create biochar-amended SCG composts. The control treatment used SCG without BC amendment. All the treatments were mixed with bacterial aliquots, and the moisture content was adjusted to 60%. The mixture (about 3 kg) was then placed into compost reactors with a hole at the bottom for leachate drainage or airflow.

Microorganisms from well-matured maize straw compost were extracted to serve as a starter for SCG, BC compost samples, which are initially sterile due to exposure to high temperatures. The mature compost was sourced from farm-based windrow composting facilities of maize straw that were curing for eight months, making it an excellent source of microorganisms. The process involved mixing 100 g of compost samples with 1 L of distilled water and shaking the mixture for 2 h to produce eluates rich in composting bacteria. The resulting solution was then evenly sprayed onto the compost materials as a composting starter.

The compost mixtures were mechanically aerated daily for the first two weeks and every third day for the following 6 weeks to ensure oxygenation and homogenization. The mixing process was carried out rapidly to reduce heat and evaporation loss. Temperatures initially reached the thermophilic range (>55 °C) and remained there for the first week. After that, temperatures gradually decreased to ambient temperatures (20–25 °C) in the following weeks. After 4 weeks of composting, the mixtures were left open during the curing stage to reach maturity. Compost samples were collected after four weeks to determine maturity and chemical characteristics.

### 2.2. Microbial Community Analysis in Compost Samples

The composted samples collected at the end of the composting experiments from the, inoculated SCG compost and inoculated SCG + BC10 were used for microbial community analysis compared to the uninoculated SCG compost. The 16S rRNA gene was amplified and analyzed using QIIME 2. Following amplification with specific primers, purification was carried out to create the library. Illumina binary indexes and adapters were added in the index PCR step using the Nextera XT index kit, followed by another round of purification. The library concentration was measured using real-time PCR (Kyratec, SC-300G, SuperCycler, Caboolture, QLD, Australia) and then diluted to 4 nM before normalization. Normalized samples were pooled together. During sequencing, fluorescence of the added base was optically observed and recorded each time a new deoxyribose nucleotide triphosphate (dNTP) was incorporated using the synthesis method. The raw data generated after sequencing were converted to FASTA format for analysis. It is important to note that the FASTA format is used to encode nucleotide or amino acid sequences, and raw sequence data in FASTA or FASTQ format can be directly used in next-generation microbiome bioinformatics platforms such as QIIME. The detailed analytical procedures were described by [9]. The sequence analysis of the compost obtained from the PCR products and the reporting of the microbial communities were performed by the laboratory of BM Labosis.

### 2.3. Physical and Chemical Analysis

For a phytotoxicity test, seed germination and germination index (GI%) were estimated using garden cress (*Lepidium sativum* L.) obtained from local market. Briefly, a solution of 2.5 g dw of compost in 25 mL of water was prepared, shaken for 2 h, then centrifuged at 3000 rpm, and filtered by Whatman 2 filtering paper. Ten seeds were placed on sterilized filter paper in a Petri dish, with three replicate dishes for each growing compost. Afterwards, 5 mL of the compost solution was added to the corresponding Petri dishes. Similarly, a control was prepared with deionized water. All the Petri dishes were incubated in the dark for 72 h at 25 °C. GI was calculated from the relative seed germination % and relative root length % (GI (%) = 100 × G/Gc × L/Lc) [15], where G and L stand for the germinated seeds and root length of the samples, respectively, and Gc and Lc stand for the corresponding values for the distilled water. For relative germination, living seedlings that germinated were counted. Similarly, for relative root length, living roots were measured.

In order to measure dissolved organic carbon (DOC), the samples were treated with ultra-pure water at a solid to water ratio of 1:10 (*dw*/*v*), using a horizontal shaker at 200 rpm for 3 h, followed by centrifugation at 3000× *g* for 20 min and filtration with 0.45 μm syringe filters. The DOC of the leaching solution was analyzed using a TOC analyzer (Shimadzu, VCSH, Kyoto, Japan).

The pH values in the compost samples were determined in water extracts obtained with a solid to liquid ratio of 1:10 (*dw*/*v*) using a pH meter (Schott CG 840, Eindhoven, The Netherlands).

To determine the degree of humification, a 1 g air-dried sample was agitated in a 50 mL, 0.5 M sodium hydroxide (NaOH) solution for 2 h, followed by centrifugation at 3000 rpm for 25 min. The absorbance of the supernatant was measured using a spectrophotometer (Hewlett–Packard Company, Wilmington, DE, USA) over a range of 190 to 1100 nm to assess the decomposition level. The optical density of the solution was recorded at 465 nm (E4) and 665 nm (E6), and the E4/E6 ratio was then determined to characterize the intensity of decomposition.

The essential plant nutrients and trace element quantifications of the composts, substrates, were conducted using an Inductively Coupled Plasma Optical Emission Spectrometry (ICP-OES) (Spectro Arcos, Kleve, Germany) after nitric (HNO_3_) and perchloric acid (HClO_4_) digestion at 200 °C [15].

For leaf chlorophyll content estimation, plant leaves that are picked directly from the plant at the end of the 30 days are used. The obtained chlorophyll content in plant leaves under the respective treatment is assessed according to the methodology of Ozdemir et al. (2021) [16], using 100 mg of fresh leaf extracted overnight with 80% acetone and centrifuged at 10,000× *g* for 5 min. Then, the solution was put into a cuvette and its absorbance was measured at wavelengths of 645, 663, and 470 nm using a UV–visible spectrophotometer (Hitachi-U2001, Tokyo, Japan).

### 2.4. Plant Cultivation Experiments

To evaluate the potential fertilizing capacity of the SCG composts, four growing media were prepared by mixing SCG-BC10 compost as fallows: commercial peat without compost (T_0_); commercial peat + 15% SCG-BC (T_1_); commercial peat + 30% SCG-BC (T_2_); commercial peat + 50% SCG-BC (T_3_); and commercial peat with fertilizer (T_4_). Garden cress (*L. sativum*) and spinach (*Spinacia oleracea* L.) were chosen for the plant production experiment because they germinate quickly, grow fast, are easy to measure, and respond to organic nutrients. The seeds were planted directly into 7 × 7 cm pots and the pots were placed on the vertical trellis growth system. The pots were manually watered daily by spraying tap water to each pot until reaching field water capacity to prevent drainage. Emergence was observed after 2 days for garden cress and after 5 days for spinach. After 30 days of emergence, the plant growth experiments were terminated and fresh leaves were collected at the end of the experiments to determine the chlorophyll content.

### 2.5. Statistical Analysis

An analysis of variance (ANOVA) was performed to assess the variations among the physical and chemical properties of the composts and their agronomic potential. When the *p*-value for the ANOVA was less than 0.05, the means were compared using Tukey’s test at a 0.05 significance level. All statistical analyses were carried out using the StatsDirect software package (V2.7.2, StatsDirect, Ltd., Altrincham, Cheshire, UK).

## 3. Results and Discussion

### 3.1. Chances in Compost Maturity Parameters

**Figure 2:**
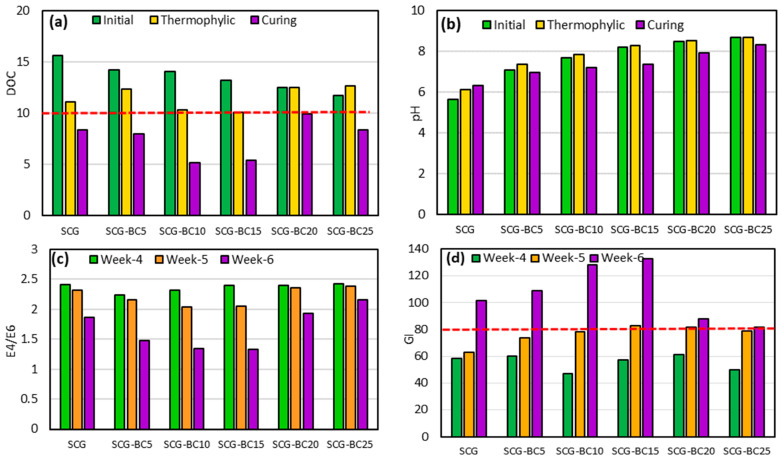
Variations in (**a**) DOC, (**b**) pH, (**c**) E4/E6, and (**d**) GI during composting of spent coffee grounds with various levels of biochar. The red line indicates DOC’s stability boundary and GI’s phytotoxicity boundary.

The dissolved organic carbon (DOC) is a key component of the organic matter pool that induces rapid decomposition, and its decrease indicates good microbial activity, effective composting, and high-quality compost [17]. Figure 2a demonstrates the impact of adding biochar on the DOC levels. The DOC content reduced from 5.63 mg kg^−1^ in SCG to 4.14 mg kg^−1^ in the treatment with 25% biochar. Furthermore, the DOC levels decreased during composting, particularly at higher biochar rates. The recommended DOC content for mature compost is 4–10 mg kg^−1^ [18]. According to these values, the addition of biochar accelerated the composting process, with biochar-amended compost reaching stability by week 4, while the SCG control compost stabilized after week 6. The porous nature of biochar decreased compost density, improved aerobic conditions, offered a habitat for microorganisms, and enhanced microbial activity, leading to DOC decomposition [17]. Moreover, the high porosity and large surface area of biochar allowed for the absorption of DOC, contributing to the decrease in its concentration. Importantly, the DOC levels observed in this study met the stability and maturity requirement (<10 g kg^−1^) as reported in previous research [18].

PH is a critical factor influencing microbial growth and is commonly used to assess compost maturity. The addition of biochar had a significant effect on pH. The pH values of SCG-BC increased from 6.34 to 8.35 when 5% or 25% biochar was added to the SCG. Despite the acidic nature of fresh SCG, the addition of biochar increased the pH of the compost, which could be related to the formation of alkali metals such as P, K, and Ca, which are abundant in poultry litter [9]. In Figure 2b, it can be seen that the pH values of SCG and SCG-BC decreased significantly during the initial phase, increased during the thermophilic phase, and finally stabilized during the curing phase. The pH of the compost was lowered during the initial rapid decomposition phase by the formation of organic acids and then stabilized by the removal of ammonia [6], the development of humic substances [19], or buffering by biochar [5] in all composts. Maintaining an appropriate pH is essential to promote effective microbial activity and the smooth running of the composting process. The standard for organic fertilizers and compost in Turkey specifies that the pH of the compost should be in the range of 5.5 to 8.5. All compost treatments reached the specified pH range after 6 weeks, indicating the maturity of the compost samples.

Spectroscopic analysis allows us to assess how mature compost is by examining the transformation properties of the organic matter. The E4/E6 ratio, measured by the optical density of compost extract solution at 465 and 665 nm, reflects the degree of humification of the compost. A low E4/E6 value means that the compost is highly mature and stabilized [18]. In the study, it was found that the E4/E6 values decreased significantly when BC was added to the compost at 10 and 15%, indicating that adding BC can improve compost maturity (Figure 2c). However, the decrease in E4/E6 values was slower at 20 and 25% BC-added compost, possibly due to the relatively high pH over 8.5. The quality of the compost was improved due to the stimulation of microbial abundance and biomass, as well as the provision of habitat by adding BC, leading to increased condensation of aromatic humus components during composting.

Germination index (GI) is a sensitive parameter used to evaluate the maturity and phytotoxicity of compost. A GI value of over 80% is necessary to meet maturity standards. At the end of the fourth week of composting, GI values ranged from 47 to 61%, indicating the presence of phytotoxic substances and immaturity of the compost (Figure 2d). By the fifth week, the GI in compost samples improved and reached the maturity threshold of 80%. After the sixth week, the GI values of the final compost ranged between 81.46% and 132.46% (Figure 2d), indicating complete maturity. The increasing GI values correlated with the rate of biochar addition, showing a peak at 15% biochar, with the highest GI value of 132.46%, a 30.48% increase compared to the SCG. This increase signifies reduced phytotoxicity, attributed to the adsorption and reduced production of phytotoxic substances due to biochar addition. This shows the efficacy of the biochar acting as a bulking agent, also being able to adsorb volatiles and toxic substances. However, at higher biochar levels of 20% and 25%, the GI value decreased (Figure 2d), likely due to the presence of harmful compounds in biochar, such as polycyclic aromatic hydrocarbons, polychlorinated biphenyls, and other toxic compounds [20].

### 3.2. Bacterial Community and Diversity

Bacteria are the predominant group in composting processes as they have the ability to attack highly complex, versatile organic substrates by releasing a broad spectrum of extracellular enzymes. Therefore, their characterization based on their diversity and richness is fundamental for the understanding of the composting process and especially for the composting of bioactive molecules such as SCG. The roasting, brewing, and cooking of SCG enriches a variety of bioactive compounds in SCG [21]. In the present study, uninoculated SCG, inoculated SCG, and inoculated SCG + BC10 compost samples were subjected to bacterial community and diversity index analysis after six weeks in the maturation phase.

**Figure 3:**
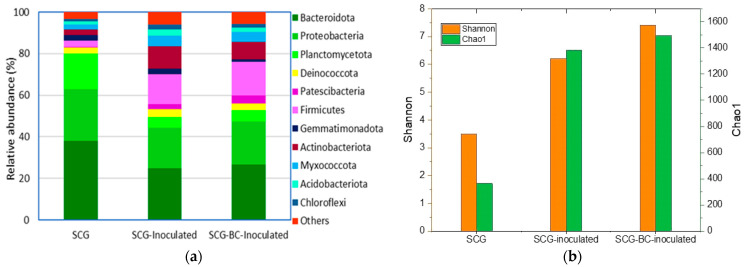
Effect of starter inoculation on compost bacterial community composition at the phylum level (**a**) and, alpha diversity indices (**b**) in spent coffee grounds and spent coffee grounds biochar at a 10% mixture.

Figure 3 illustrates the impact of starter inoculation on the bacterial community composition at the phylum level and the alpha diversity indices in spent coffee grounds and spent coffee grounds biochar at a 10% mixture. At the end of composting, the compost treatments showed different relative abundance of bacterial communities at the phylum level. There were five dominant phyla in the studied treatments: *Bacteroidota* (25–37%), *Proteobacteria* (19–25%), *Planctomycetota* (5–17%), *Firmicutes* (3–16%), and *Actinobacteriota* (2–11%) (Figure 3a), which is common for compost experiments [22]). However, the five dominant strains were detected in all treatments. Previous studies showed that the sequence of the bacterial community during the composting process is as follows: *Proteobacteria* in the initial phase, *Firmicutes* in the thermophilic and mesophilic phases, and *Bacteroidota* in the maturation phase [23]. On the other hand, inoculation with compost starter increased the relative abundance of *Firmicutes* and *Actinobacteriota* in both the SCG and SCG-BC composting processes, which replaced the *Bacteroidota* and *Planctomycetota* compared to the non-inoculated treatment. These dominant bacterial phyla collectively have a strong ability to decompose organic waste and are usually the most abundant phylum in organic-rich environments [9]. However, the presence of *Firmicutes* and *Actinobacteriota* in higher abundance in the inoculated treatments could apparently be due to a more mature compost. *Firmicutes* and *Actinobacteriota* are prevalent in mature corn straw compost [24], suggesting that the success of starter inoculation and the degradation and maturity it promotes in the final compost. The presence of *Patescibacteria*, which are capable of hydrolyzing phenolic compounds [25], in the inoculated treatments compared to the non-inoculated compost, indicates the effectiveness of the compost starter. In addition, the presence of *Actinobacteria* in the maturation phase indicates a high conversion of lignocellulose to humus [14].

The SCG is a rich source of bioactive compounds, such as phenolics (i.e., chlorogenic, gallic, caffeic, quinic, ellagic, cinnamic, coumaric, and ferulic acids), alkaloids (i.e., caffeine, trigonelline), and flavonoids (i.e., catechin, epicatechin, rutin, quercetin, and naringin) [26]. These compounds possess potent biological properties and can function as antimicrobial, antifungal, and antiviral agents [11]. Thus, it is crucial to promote the proliferation of active microbial communities during composting, as these communities play a vital role in neutralizing harmful substances present in SCG. Alpha diversity is utilized to assess the richness and relative abundance of bacteria in compost samples (Figure 3b). The bacterial community diversity and richness of mature compost were notably higher in SCG and SCG + BC. This increase in diversity and richness of the bacterial communities was attributed to bacterial inoculation [27]. The Chao1 index serves as an estimator of species richness, reflecting the total number of distinct species in each sample. Microbial inoculation significantly elevated the Chao1 values in SCG and SCG-BC compost, indicating a greater species richness. SCG-BC exhibited the highest Chao1 value (1496), followed closely by SCG (1454), suggesting that bacterial inoculation significantly boosted microbial species richness. Furthermore, the Shannon index evaluates species diversity and evenness within each sample, with higher values indicating a more balanced distribution of species abundance [28]. SCG-BC and SCG displayed relatively higher Shannon index values (7.40 and 6.20, respectively) compared to uninoculated SCG (3.49), suggesting a more balanced representation of microbial species in inoculated composts [29]. The Shannon diversity index integrates species richness and evenness, with higher Shannon values indicating increased diversity and evenly distributed microbial communities in compost samples. Overall, the alpha diversity results indicate that bacterial inoculation derived from mature compost significantly impacts the creation of diverse microbial communities in SCG and BC that initially did not contain microorganisms. According to the Shannon and Chao1 index, both SCG and SCG-BC exhibited higher diversity than uninoculated SCG, indicating a more varied microbial composition. Therefore, it is possible to expect more rapid composting, as well as the removal of bioactive compounds in spent coffee grounds that have a negative impact on compost maturity and phytotoxicity parameters.

### 3.3. Effects of Biochar on Nutrient Contents of Composting Products

The variation in essential plant nutrients in the final compost product affected by biochar addition are shown in Table 2. In this study, the content of TN of the final BC-compost products were 2.21–2.88%, which is significantly smaller than the TN content of the SCG compost without biochar amendment (3.20%). Some previous studies showed that the TN contents of the final compost depends on type and rate of bulking agent mixed to compost. Some studies also report that biochar addition had a significant effect on the TN content due to preserving TN in compost [15]. The response of final compost TN to biochar addition was significantly influenced by composting conditions, such as compost method, initial carbon to nitrogen ration, and initial moisture content [30], which may vary dramatically among different material and studies.

In the presence of biochar, the total phosphorus and potassium contents in compost increased from 0.20 to 0.53%, 0.49 to 0.84%, respectively, and significantly higher than with sole SCG control compost (Table 2). This increase could be attributed to the higher levels of P and K in poultry litter BC (Table 1). Previous studies also indicated that compost mixed with biochar can effectively boost essential plant nutrients [31,32]. Compost users criticized the low nutrient content of composts, stating that a large amount of compost is required to meet plant requirements [9]. In this context, nutrient-rich poultry litter BC as a compost additive can satisfy user expectation. Moreover, there is evidence suggesting that organic nutrient sources, when applied at maximum yields, can lead to organic build-up, acting as a slow-release fertilizer due to beneficial microbial action, thus promoting plant growth [15].

The impact of adding biochar on the levels of major plant nutrients N, P, and K is crucial for the quality and potential applications of compost products to cultivated crops. When 10% biochar was added to the final composting product, the P content almost doubled compared to the sole SCG compost, and the K content similarly increased almost twofold. However, the addition of biochar led to a reduction in TN content at each increased level of added BC, ranging from 2.88 to 2.21% (Table 2). This effect is likely due to biochar addition improving the P and K in compost mixture while mitigating N loss through volatilization, attributed to its favorable structure and aeration of the compost substrate, driven by characteristics such as porosity, large specific surface area, and functional nutrient binding groups [13]. Furthermore, alongside the enhancement of plant nutrients, the desired promotion of nutrient mineralization dynamics by microbial enzymes in the compost matrix is typically sought to enhance compost quality and nutrient availability [9]. Therefore, these results confirm the hypothesis of this study that composted SCG can be used as a low-cost and environmentally friendly bio-nutrient resource due to its high nitrogen content and sufficient essential macro and micro plant nutrients.

### 3.4. Leafy Vegetable Pigment Contents

The chlorophyll content in leaves serves as a significant indicator of photosynthetic activity, which in turn contributes to enhanced plant growth as a result of the positive response to vital plant nutrients provided by applied composts [16]. The levels of chl-a, chl-b, and total chlorophyll in garden cress leaves increased with the application of increasing amounts of SCG-BC compost in the growing media (Figure 3a). Notably, plants fertilized with inorganic fertilizer exhibited the highest chl-a and total chlorophyll content, with no significant difference observed compared to compost-treated plants at 30 and 50% compost mixtures. Conversely, unfertilized plants showed the lowest chlorophyll content. The increased leaf chlorophyll content, compared to the control group, may be attributed to the improved nutrient supply provided by the nutrient pool of SCG-BC [33], thereby indirectly indicating enhanced photosynthetic activity. Garden cress, being a leafy vegetable, is particularly receptive to nutrients such as nitrogen, and organic amendments are effective in providing nutrients due to their slow-release properties, as opposed to inorganic nutrients [12].

**Figure 4:**
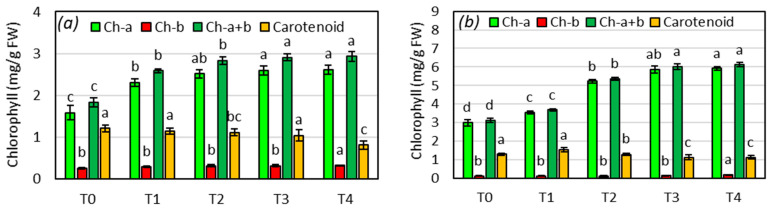
Chlorophyll and carotenoid contents of *L. sativum* (**a**) and *S. oleracea* (**b**) grown in increasing amounts of SCG + BC compost. Values are means for triplicate samples. Different lowercase letters on the histogram of the same color indicate significant differences at *p* < 0.05.

The levels of chlorophyll a and b, as well as the total chlorophyll (Chl a + b) content in spinach leaves per unit of fresh weight, were significantly influenced by the treatments (*p* < 0.01), indicating a positive response of spinach to the increasing dose of SCG + BC compost. Specifically, the Chl-a content in the leaves was significantly higher in the fertilized treatment (Figure 4b). Among the treatments, T_3_ (50% compost) resulted in the highest chlorophyll content in spinach leaves compared to the other compost treatments, which received 15 and 30% compost. These findings demonstrate that the nutrient content and mineralization rate support spinach growth without the need for additional mineral fertilizer, which is crucial for sustainable leafy vegetable cultivation in the short term and reducing the environmental impact of excessive use of inorganic fertilizers. Numerous previous studies demonstrated the sufficiency of organic nutrient sources in potted cultivation due to the improved nutrient use efficiency [34], although some studies indicated the necessity of additional inorganic fertilizers to support plant growth and achieve higher yields [35]. This may be attributed to the limited mineralization rate of organic nutrients from bio-nutrient resources [36]. However, in this study, the nutrient mineralization plant-providing rate of SCG and the nutrient retention capacity of BC proved to be effective in meeting the plant’s nutrient demands during a one-month growth cycle. Nonetheless, longer crop growth cycles may require additional fertilizer, as suggested in previous studies that recommend combining compost with fertilizer to maintain optimum tissue nutrient levels, leaf chlorophyll content, and ultimately achieve higher yields [9,18].

## 4. Conclusions

The outcomes of composting experiments indicate that it is possible to produce nutrient-rich compost suitable for crop fertilization by combining spent coffee grounds and poultry litter biochar at a 10% ratio and introducing the mixture to starter inoculum obtained from maize straw compost. The incorporation of biochar notably increased the abundance, richness, and diversity of microorganisms involved in composting, including *Patescibacteria*, which are crucial in breaking down phenolic compounds found in spent coffee grounds and biochar. Moreover, the biochar enriched the compost with phosphorus and potassium while preserving nitrogen content. Additionally, increasing the amount of compost in growing media led to a significant rise in chlorophyll content in the leaves of garden cress and spinach during a 30-day growth period. Taken together, the findings suggest that the compost derived from spent coffee grounds and biochar is well-suited for enhancing the growth of garden cress and spinach, providing effective nutrients for these leafy vegetables, and facilitating soil–plant–atmosphere nutrient cycling. It is expected that composting spent coffee grounds with biochar could produce nutrient-rich biofertilizer in line with a circular economy strategy, offering a high-quality organic fertilizer suitable for the cultivation of leafy vegetables in agriculture. Returning waste organic resources to the soil can help build up carbon, reduce reliance on chemical inputs, and contribute to farmer welfare while minimizing waste accumulation and protecting the environment.

## Figures and Tables

**Table 1:** Properties of raw spent coffee ground and poultry litter biochar as received form.

Parameters	Spent Coffee Ground	Biochar
Moisture content (%)	63.62	10.26
Ash content (%)	3.15	35.82
Volatile matter (%)	96.85	51.43
pH	5.61	10.25
C %	52.06	52.25
H %	5.87	2.04
N %	2.16	1.27
S %	0.20	0.05
O %	58.17	44.39
C/N	24.08	23.12

**Table 2:** Nutrient content of increased dose of biochar added spent coffee ground composts (mean ± sd).

Parameter	SCG	SCG-5%BC	SCG-10%BC	SCG-15%BC3	SCG-20%BC	SCG-25%BC
TN (%)	3.20 ± 0.43 a	2.88 ± 0.22 b	2.72 ± 0.11 c	2.61 ± 0.22 d	2.46 ± 0.04 e	2.21 ± 0.02 f
TP (%)	0.20 ± 0.02 f	0.28 ± 0.02 e	0.39 ± 0.01 d	0.42 ± 0.02 c	0.47 ± 0.04 b	0.53 ± 0.01 a
TK (%)	0.49 ± 0.03 f	0.54 ± 0.01 e	0.66 ± 0.06 d	0.71 ± 0.02 c	0.79 ± 0.01 b	0.84 ± 0.02 a
Ca (%)	0.14 ± 0.05 f	0.18 ± 0.03 e	0.24 ± 0.04 d	0.32 ± 0.02 c	0.37 ± 0.03 b	0.43 ± 0.03 a
Mg (%)	0.10 ± 0.02 e	0.11 ± 0.02 d	0.13 ± 0.01 d	0.16 ± 0.02 c	0.19 ± 0.01 b	0.21 ± 0.01 a
S (%)	0.01 ± 0.01 d	0.02 ± 0.01 c	0.02 ± 0.01 c	0.03 ± 0.01 b	0.03 ± 0.05 b	0.04 ± 0.04 a
Fe (mg kg^−1^)	124 ± 1.21 f	136 ± 1.21 e	144 ± 1.21 d	149 ± 1.21 c	153 ± 1.71 b	157 ± 1.23 a
Mn (mg kg^−1^)	58 ± 0.04 f	64 ± 0.89 e	67 ± 1.01 d	72 ± 0.82 c	76 ± 1.03 b	78 ± 0.92 a
Zn (mg kg^−1^)	85 ± 0.91 f	88 ± 0.79 e	92 ± 0.85 d	95 ± 0.87 c	97 ± 0.92 b	103 ± 0.84 a
Cu (mg kg^−1^)	26 ± 0.68 f	40 ± 1.15 e	43 ± 0.93 d	46 ± 1.33 c	50 ± 0.86 b	52 ± 1.44 a
B (mg kg^−1^)	14 ± 0.01 c	15 ± 0.24 c	15 ± 0.34 c	17 ± 0.37 b	17 ± 0.22 b	19 ± 0.51 a

For each row, different lowercase letters indicate significant differences among treatments according to the Tukey test (*p* < 0.05).

## Data Availability

All data are reported in the manuscript.
